# The activation of group II metabotropic glutamate receptors protects neonatal rat brains from oxidative stress injury after hypoxia-ischemia

**DOI:** 10.1371/journal.pone.0200933

**Published:** 2018-07-25

**Authors:** Ewelina Bratek, Apolonia Ziembowicz, Agnieszka Bronisz, Elzbieta Salinska

**Affiliations:** 1 Department of Neurochemistry, Mossakowski Medical Research Centre, Polish Academy of Sciences, Warsaw, Poland; 2 Harvey Cushing Neuro-Oncology Laboratories, Department of Neurosurgery, Brigham and Women’s Hospital, Harvard Medical School, Boston, Massachusetts, United States of America; Doheny Eye Institute/UCLA, UNITED STATES

## Abstract

Birth asphyxia resulting in brain hypoxia-ischemia (H-I) can cause neonatal death or lead to persistent brain damage. Recent investigations have shown that group II metabotropic glutamate receptor (mGluR2/3) activation can provide neuroprotection against H-I but the mechanism of this effect is not clear. The aim of this study was to investigate whether mGluR2/3 agonists applied a short time after H-I reduce brain damage in an experimental model of birth asphyxia, and whether a decrease in oxidative stress plays a role in neuroprotection. Neonatal H-I in 7-day-old rats was used as an experimental model of birth asphyxia. Rats were injected intra peritoneally with mGluR2 (LY 379268) or mGluR3 (NAAG) agonists 1 h or 6 h after H-I (5 mg/kg). The weight deficit of the ischemic brain hemisphere, radical oxygen species (ROS) content levels, antioxidant enzymes activity and the concentrations of reduced glutathione (GSH) were measured. Both agonists reduced weight loss in the ischemic hemisphere and mitigated neuronal degeneration in the CA1 hippocampal region and cerebral cortex. Both agonists reduced the elevated levels of ROS in the ipsilateral hemisphere observed after H-I and prevented an increase in antioxidant enzymes activity in the injured hemisphere restoring them to control levels. A decrease in GSH level was also restored after agonists application. The results show that the activation of mGluR2 and mGluR3 a short time after H-I triggers neuroprotective mechanisms that act through the inhibition of oxidative stress and ROS production. The prevention of ROS production by the inhibition of glutamate release and decrease in its extracellular concentration is likely the main mechanism involved in the observed neuroprotection.

## Introduction

Birth asphyxia resulting in hypoxic-ischemic brain damage remains an important problem in modern obstetrics, even in developed countries. In resource-rich countries, severe perinatal asphyxia (causing death or severe neurological impairment) accounts for 10% to 20% of problematic delivery cases [1,2]. The encephalopathy that follows a hypoxic-ischemic insult reflects an evolving process characterized by an initial primary injury followed by a self-sustaining cascade of harmful biochemical events that lead to further brain damage.

An increased release and extracellular retention of glutamate, which leads to an excessive activation of ionotropic glutamate receptors, especially N-methyl-D-aspartate (NMDA) receptor (excitotoxicity), and the accumulation of toxic products such as reactive oxygen species (ROS) resulting from insufficient oxygen and glucose supply are the most important factors involved in hypoxia-ischemia brain injury. The accumulation of ROS shifts the balance between oxidants and antioxidants in favour of oxidants, thus initiating oxidative stress. Cells are equipped to combat oxidative stress and to neutralize reactive oxygen species; superoxide dismutase (SOD), catalase (CAT) and glutathione peroxidase (GPx) supported by glutathione are well known enzymes that eliminate ROS [3].

The neuroprotective effect of antagonists of NMDA and α-amino-3-hydroxy-5-methyl-isoxazole-4-propionic acid (AMPA) ionotropic glutamate receptor subtypes have been demonstrated in numerous animal models of cerebral hypoxia-ischemia; however, the observed side effects of these compounds preclude their clinical use [4].

Metabotropic glutamate receptors (mGluR) and the role of individual mGlu receptor subtypes in the pathophysiology of human disorders have been a subject of attention for several years. Research has shown that they play a role in schizophrenia, depression, drug addiction, anxiety, Alzheimer’s disease, Parkinson’s disease and ischemic brain damage [5–8].

Recently, the role of group II metabotropic glutamate receptors in hypoxic-ischemic (H-I) brain injury has gained increasing attention. Negatively coupled to cyclic AMP formation mGluR2 and mGluR3, which act as regulating glutamate transmission presynaptic autoreceptors, appear to serve as promising targets for inducing neuroprotection. Group II mGluRs are localized along the periphery of the synaptic cleft and respond to any excessive glutamate that escapes from the synaptic active zone; activated receptors suppress this release [9,10].

It has been shown that mGluR3 are also expressed by astrocytes and glia and that their activation enhances glutamate uptake [11,12].

The neuroprotective effect of selective agonists of mGluR2/3 against ischemic brain injury has been shown through a number of animal studies. N-acetylaspartylglutamate (NAAG), a full agonist of mGluR3, has been shown to be protective in focal cerebral ischemia and in the neonatal rat model of hypoxia-ischemia [13,14], while (-)-2-oxa-4-aminobicyclo[3.1.0]hexane-4,6-dicarboxylic acid (LY379268), a mGluR2/3 agonist exhibiting higher modulation potential towards mGluR2, has been reported to serve as a neuroprotective factor in the same model of hypoxia-ischemia and experimental global ischemia in gerbils [15,16]. The mechanisms of this neuroprotection remain not fully understood and appear to be complex. The reduction of glutamate release appears to serve as an obvious element of the neuroprotection mediated by mGluR2/3 activation; however, a body of evidence shows that this is not the only neuroprotective activity of group II mGluRs. Bruno et al. [17] suggested that the neuroprotection observed after the activation of mGluR3 is mediated by the synthesis and release of transforming growth factor- beta (TGF-β) from astrocytes. Other mechanisms that have been proposed involve the enhancement of glutamate uptake, inhibition of programmed cell death and protection from oxidative stress [12,16,18].

Although the relationship between mGluRs and oxidative stress was demonstrated many years ago, there are relatively few studies have investigated the role of mGluR2/3. It has been shown that the activation of group II mGluRs protects neurons from glucose-induced oxidative injury [19], attenuates oxidative stress-induced cell death in spinal cord injuries [20] and reduces ROS production in immature rat brain during seizure induced by the bilateral intracerebroventricular infusion of DL-homocysteic acid [21]. It has also been recently shown that the activation of group II mGluRs protects against the ischemia-induced free radical programmed death of rat brain endothelial cells, although this neuroprotective effect is considered to reflect a complex metabotropic glutamate receptors response [22]. The exact mechanisms of mGluR2 and mGluR3 interaction with ROS production and inactivation are not clear, though Berent-Spillson and Russell [19] have shown that the activation of mGluR3 increases the concentration of glutathione, which is an important element of antioxidant cell defence. However, there is no available information on the effect of group II mGluRs activation on changes in antioxidant enzymes activity resulting from ischemic insults.

Assuming that the activation of group II mGlu receptors shortly after H-I has a neuroprotective effect, we wanted to more closely investigate the molecular mechanism(s) of this effect.

The aim of our study was to investigate whether the activation of mGluR2/3 within a short time after experimental birth asphyxia in 7-day old rats will result in neuroprotection and whether the antioxidant mechanisms are engaged in this process.

## Materials and methods

### Ethics approval and consent for participation

All experiments described in this paper were approved by the 4th Local Ethical Committee based in Warsaw, Poland, and were performed in accordance with Polish governmental regulations (Dz.U.97.111.724), and with the European Community Council Directive of 24 November 1986 (86/609/EEC) and with Directive 2010/63/EU. All surgeries were performed under halothane anaesthesia and all efforts were made to minimize animal suffering and the number of animals used.

### Induction of cerebral hypoxia—ischemia

Neonatal cerebral H-I was induced according to Rice et al. [23]. Birefly, 7-day-old Wistar rat pups of both sexes (weight 12–18 g) were anaesthetized with isoflurane (4% for induction, and 1.5–2.0% for maintenance) in a mixture of nitrous oxide and oxygen (0.6:1). The left common carotid artery was exposed and cut between double ligatures of silk sutures, or was only exposed (sham control). Before closing, the wound was treated with local anesthetic (lignocainum). After 60 min of recovery animals were placed in a humidified chamber (35°C) and exposed to a hypoxic gas mixture (8% oxygen in nitrogen) for 75 min. This duration of hypoxia-ischemia is typically associated with infarction predominantly of the cerebral hemisphere ipsilateral to the carotid artery occlusion [24,25]. After hypoxic treatment the animals were returned to the cages and housed with their mother at room temperature (22 °C) over a 12:12 h light—dark cycle with ample food and water. The condition of animals, which stayed in an experiment for fourteen days, was controlled twice a day and, if necessary, an anesthetic was applied locally.

### Drug application

Animals were injected intraperitoneally (i.p.) with specific agonists of either mGluR2 (LY 379268) or mGluR3 (N-acetylaspartylglutamate, NAAG). Injections were made 1 h, 6 h or 24 h (evaluation of ipsilateral hemisphere weight deficit) after H-I at a dose of 5 mg/kg of body weight. These doses were determined based on previously published findings [13,15]. To investigate potential additive effects of the agonists used, one group was injected with both LY379268 and NAAG 1 h after H-I. Sham operated and H-I control rats were injected with saline.

### Evaluation of brain damage

Fourteen days after H-I the pups were anaesthetized with a lethal dose of morbital and decapitated. Brains were removed and both cerebral hemispheres were weighed. Brain damage was reflected by a deficit in the wet weight of the ipsilateral (left) ischemic hemisphere and is expressed as a percentage of the wet weight of the contralateral (right) control hemisphere.

A histochemical evaluation of brain damage was performed on brains isolated 7 days after H-I. Animals were anaesthetized with halothane and subjected to intracranial perfusion fixation with 4% neutralized formalin (Sigma-Aldrich, St. Louis, Missouri, USA). The brains were then removed, postfixed for 4 h in the same fixative and embedded in paraffin. Paraffin blocks containing brain tissue were cut from the area containing tissue located +2.3 to +1.6 mm from the vertical reference plane [26] into serial sections of 10 μm thick using a rotator microtome (Hydrax M 25, Zeiss). The sections were stained according to the Nissl protocol with 0.5% Cresyl violet for the histological assessment of neuronal cell damage.

Other sections of each group were analysed for apoptosis assessment. Terminal deoxynucleotidyl transferase-mediated dUTP nick end labeling (TUNEL) staining was performed to detect DNA fragmentation in cell nuclei using an In Situ Cell Death Detection Kit (Roche, Switzerland). Sections were deparaffinized in xylene, dehydrated in a graded ethanol series to distilled water, rinsed in 3% hydrogen peroxide, and incubated in 20 μg/ml proteinase K for 15 min at room temperature. Subsequently, the slides were incubated in TUNEL reaction mixture for 1 h at 37°C. Sections were analysed through a fluorescence microscope. TUNEL-positive nuclei with chromatin condensation and fragmented nuclei were considered as probable apoptotic cells. The number of TUNEL positive cells was counted in the cortex in the visual field under 200 x magnification (250 μm x 250 μm) and in a CA1 area of 100 μm in length using the AxioVision imaging program (Carl Zeiss, Aalen, Getmany).

### Determination of ROS level

The ROS levels in brain hemispheres were determined using 2,7 –dichlorofluorescein diacetate (DCF-DA), which is a fluorogenic dye that upon coming into contact with ROS is converted into highly fluorescent 2’, 7’–dichlorofluorescin (DCF) detected by fluorescence spectroscopy (ROS Detection Cell-Based Assay Kit, Cayman Chemical, USA) [27]. DHE has been shown to be oxidized by superoxide or through non-specific oxidation by other sources of reactive oxygen species.

Brains were collected 3 h after the injection of the mGluR2/3 agonists and tissues from the left and right hemispheres were homogenized separately in ice-cold 40 mM Tris-HCL buffer (pH.7.4). The resulting brain homogenates were incubated with DCF-DA (25 μM) for 30 min at 37°C. The formation of the fluorescent product DCF was monitored using a fluorescence spectrometer with an excitation wavelength of 488 nm and an emission wavelength of 525 nm. The relative fluorescence unit (RFU) was calculated per 1 mg of protein in the homogenate. The protein concentration was determined according to Bradford [28]. The effect of H-I and used agonists was estimated through the determination of the ROS level in individual brains and then standardized for variation at the basal level measured in brains from sham operated animals.

### Determination of glutathione concentration

Glutathione concentrations were measured from tissues isolated from both the left and right hemispheres 3 h after the agonists injection. The left and right hemispheres were homogenized separately in 25 mM HEPES, pH 7.4, containing 250 mM sucrose and then centrifuged at 1,000 x g for 5 min at 4°C. Reduced glutathione concentrations were determined from supernatants using a Glutathione Assay Kit (Cayman Chemical Company, USA) following the manufacturer’s procedures.

### Determination of antioxidant enzymes activity

Brains were collected 3 h after the agonists injection and the left and right hemispheres were homogenized separately in an ice cold 50 mM potassium phosphate buffer containing 1 mM EDTA (pH 7.4), and centrifuged at 10,000 x g for 15 min at 4°C. Then the supernatant was collected. Catalase activity was determined from the supernatants using a Catalase Assay Kit (Cayman Chemical Company, USA) following the manufacturer’s procedure.

For SOD activity measurements brain tissues were rinsed with PBS buffer, pH 7.4, to remove any blood cells and clots. The left and right hemispheres were homogenized separately in cold 20 mM HEPES buffer, pH 7.2, containing 1 mM EDTA, 210 mM mannitol, and 70 mM sucrose (5 ml per gram tissue). Homogenates were centrifuged at 1,500 x g for 5 min at 4°C. The supernatant was collected and the total activity of all types of SOD present in homogenate was determined using a Superoxide Dismutase Assay Kit (Cayman Chemical Company, USA), following the manufacturer’s manual.

Glutathione peroxidase (GPx) activity was measured both brain hemispheres separately. Tissue was homogenized in cold buffer (50 mM Tris-HCL, pH 7.5, 5 mM EDTA and 1 mM DTT) and centrifuged at 10,000 x g for 15 min at 4°C, and the supernatant was collected for assaying. GPx activity was measured using the Glutathione Peroxidase Assay Kit (Cayman Chemical Company, USA).

### Statistical analysis

The results are expressed as the means ± SEM for each experimental group. A statistical analysis of the brain damage data was performed via a paired t test. A statistical analysis of the remaining data was performed through an one way ANOVA, with a further analysis involving a post-hoc least significance test for significant differences between groups (GraphPad Prism, version 5.01; GraphPad Soft-ware Inc., La Jolla, California, USA). Differences were considered significant when *p* values of less than 0.05 were found.

## Results

### The effect of group II mGluR agonists on H-I evoked brain damage

**Fig 1:**
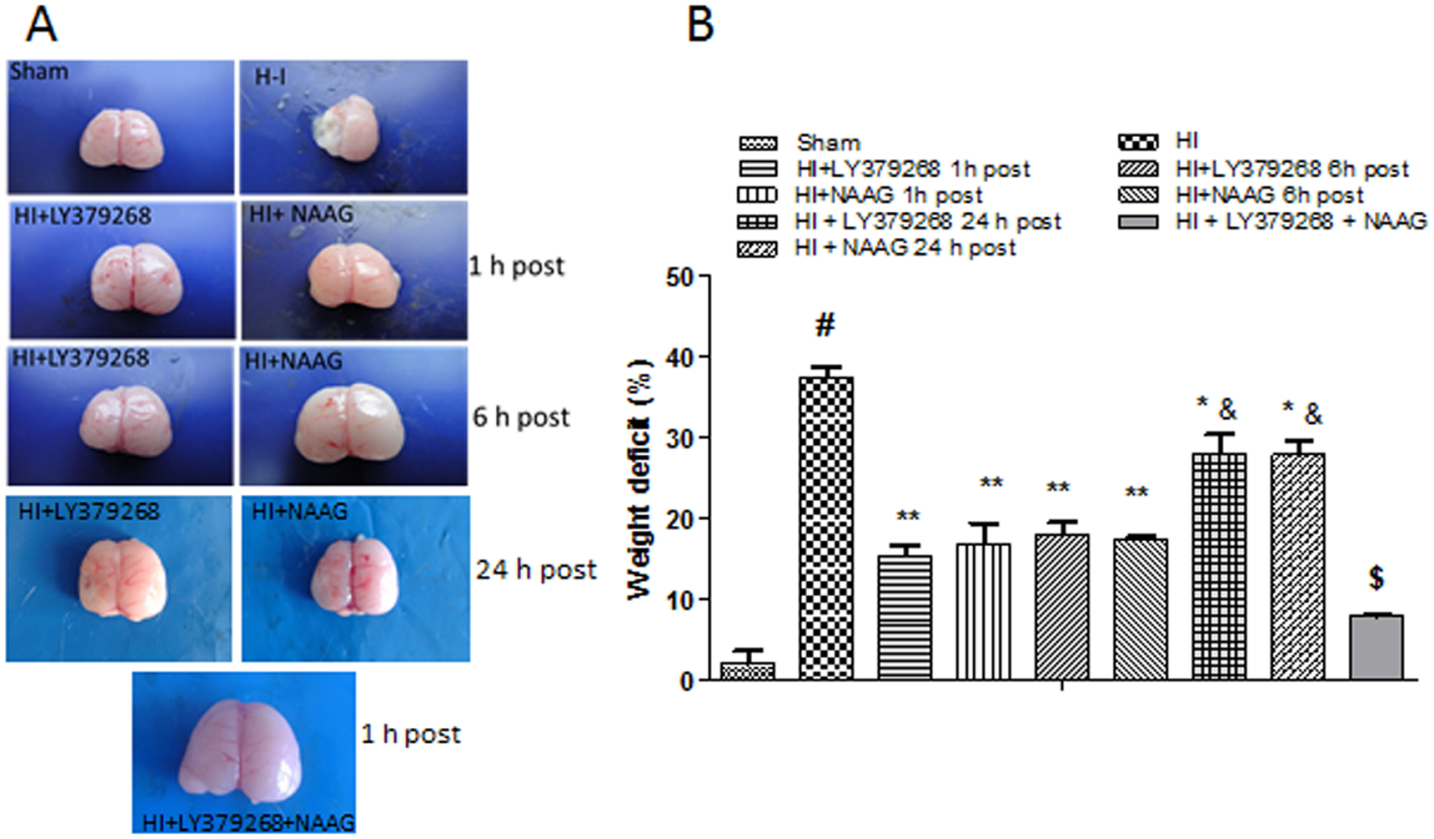
The effect of LY379268 (mGluR2) or NAAG (mGluR3) applied 1 h, 6 h or 24 h after H-I insult and agonists applied together 1 h after H-I on ipsilateral hemisphere weight loss. The weight deficit was expressed as the percentage of the contralateral (left) hemisphere. Results are presented as the mean values ± SEM, n = 3–5. Statistically significant differences: * *p* < 0.01.

H—I generated brain injury is expressed as a weight deficit in the ipsilateral brain hemisphere. Two weeks after insult the weight deficit accounted for 37% of the contralateral hemisphere (Fig 1). Intraperitoneal injections of mGluR2 and mGluR3 agonists (LY379268 and NAAG respectively) in a short time after H-I produced significant neuroprotection. LY379268 applied 1 h or 6 h after H-I resulted in a significant reduction in weight loss to 15% and 18% respectively (F_2,10_ = 69.7; *p* < 0.001), and the application of NAAG decreased weight loss to 16% and 17% respectively (F_2,10_ = 44.7; *p* < 0.001). Agonists application 24 h after H-I also resulted in a significant decrease in brain weight loss relative to patterns found found for the H-I groups, reaching a value of 28% for both agonists. However, this decrease was significantly less pronounced than that observed after agonists application at 1 h or 6 h after H-I. We found no statistically significant difference between the two agonist groups. The application of both agonists 1 h after H-I resulted in a significant reduction in ipsilateral hemisphere weight loss (6%, F_1,5_ = 38.2, p < 0.001). The decrease in weight loss observed was less significantly than that observed for groups injected with each agonist separately. This may indicate on additive neuroprotective effect of the used agonists.

Both agonists applied to sham operated animals did not produce any changes in brain weight compared to sham controls (data not shown).

For further experimentation, to investigate the molecular mechanism of neuroprotection, we have chosen time when the neuroprotective effect of agonists application was strongly manifested, which is 1 h and 6 h after H-I.

**Fig 2:**
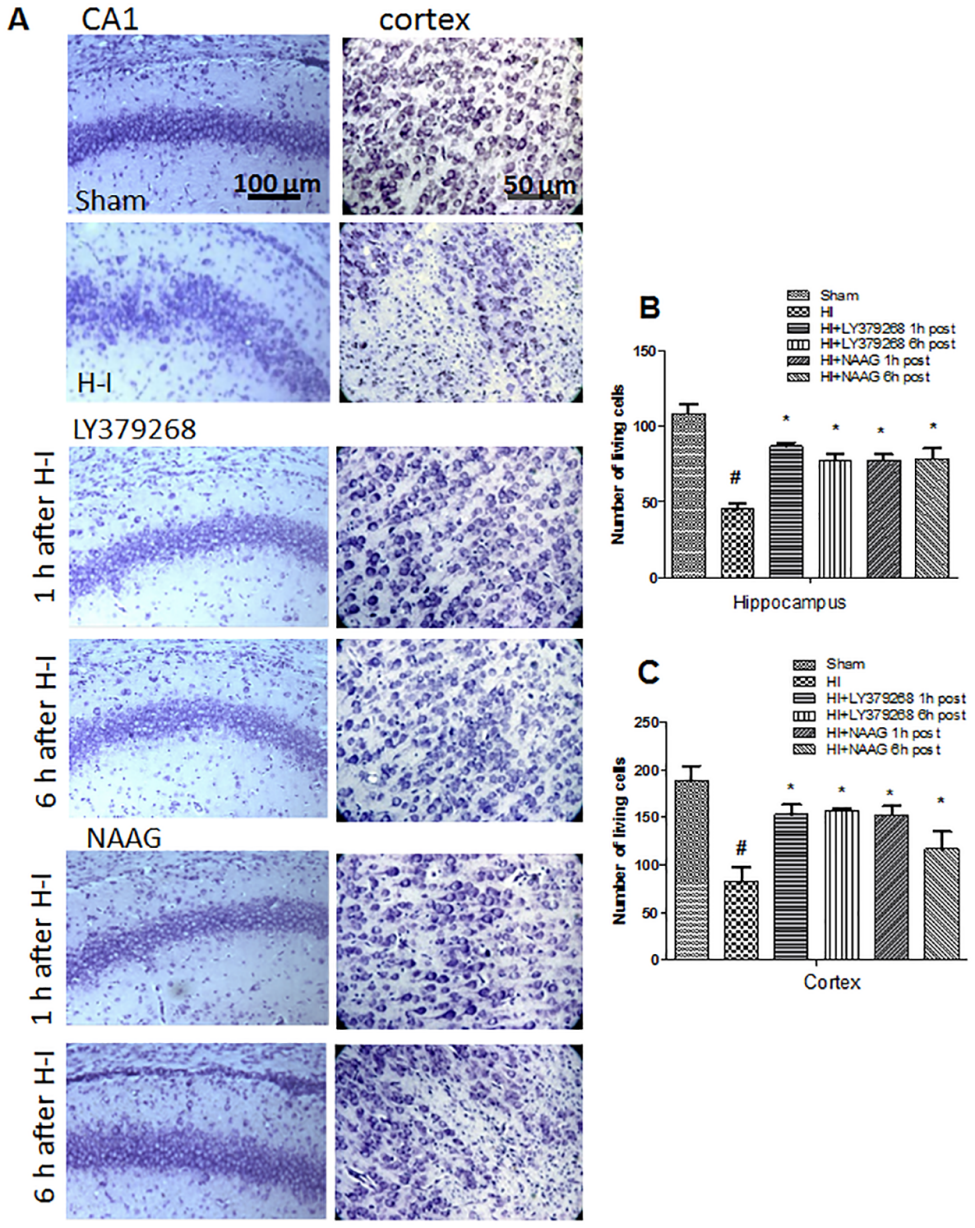
Neuroprotective effect of mGluR2 and mGluR3 agonist application observed 7 days after H—I in the CA1 region of the hippocampus and in the cerebral cortex (A). Quantification of surviving neurons in the hippocampal central CA1 region of 100 μm length (B) and in cortex—area 250 μm x 250 μm (C). LY 379268 and NAAG were applied i.p. 1 h and 6 h after H-I. Microphotographs show the hemisphere ipsilateral to H—I.

The cresyl violet staining results show that H-I evoked marked damage and disorganization of neurons in the CA1 region of the hippocampus. Cell loss in the cortex and hippocampal CA1 region observed after H-I reached 57% and 59% respectively (Fig 2). The application of LY379268 1 h and 6 h after H—I prevented changes in the CA1 region of the hippocampus and significantly decreased neuronal loss in the cortex and hippocampus. The neuroprotective effect of NAAG was more pronounced when this agonist was applied 1 h after H-I; application 6 h after H-I only partially reduced neuronal loss in the cortex and disorganization of neurons in the CA1 region of the hippocampus.

**Fig 3:**
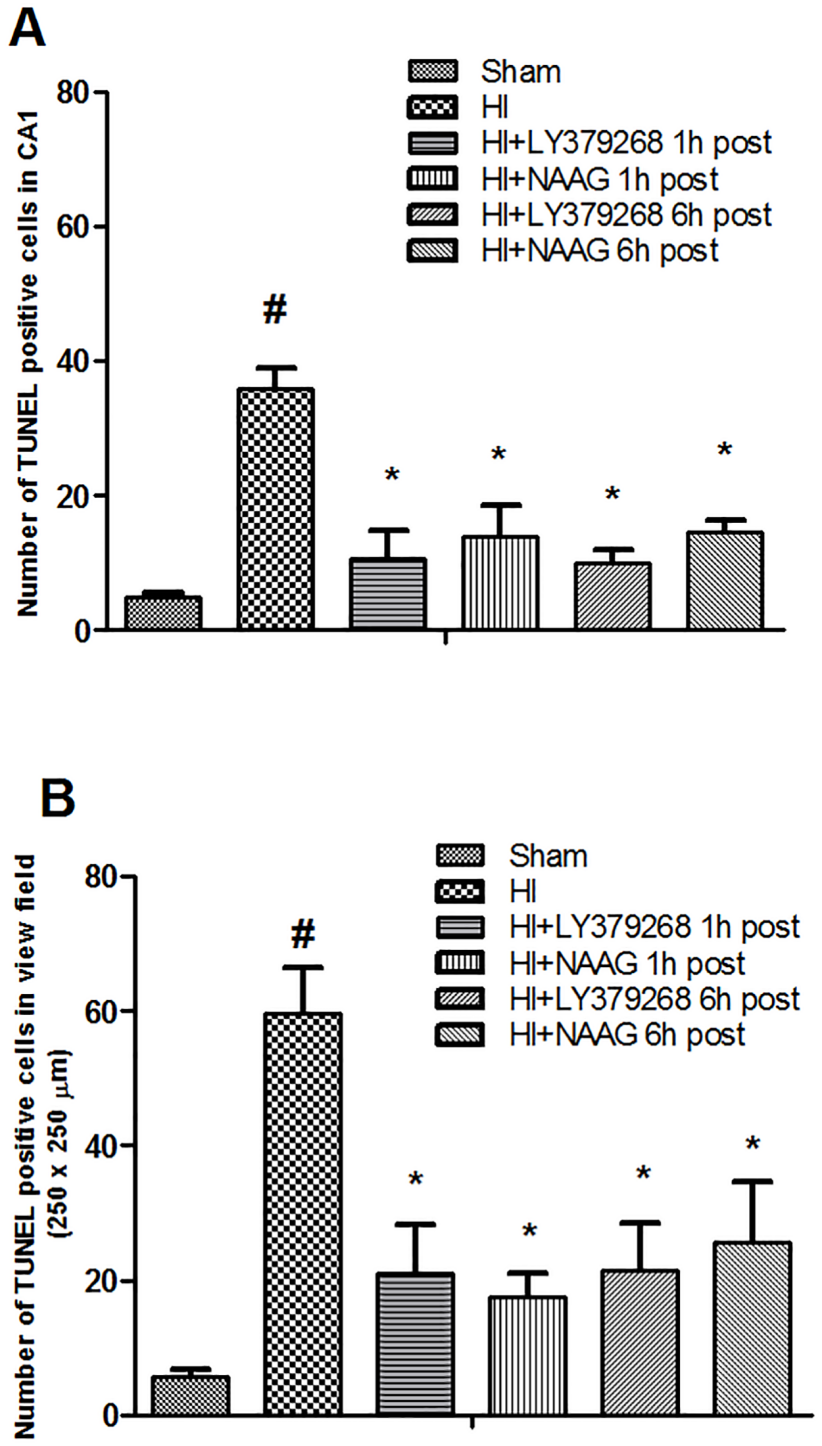
The effect of LY379268 and NAAG on the development of apoptosis in the ipsilateral brain hemisphere after H-I: (A) number of TUNEL positive cells detected in the central CA1 area of 100 μm length. (B) number of TUNEL positive cells in the cortex counted in the visual field (250 μm x 250 μm). Number of animals per group n = 3–5. Results are presented as mean values ± SEM. #—different from the sham operated group, *p* < 0.05; *—different from the H-I group, *p* < 0.01.

H-I initiated apoptotic processes and one week after the insult 36 ± 4 and 59 ± 10 TUNEL positive cells were observed in examined area of the CA1 region of hippocampus and cortex respectively (Fig 3). The application of LY379268 or NAAG 1 h after H-I reduced the number of TUNEL-positive cells observed in the central CA1 are by 70% and 61%, respectively (*p* < 0.05). The number of TUNEL positive cells in the cortex observed in the visual field was reduced by 65% and 71% respectively (*p* < 0.02 and p < 0.01). LY379268 applied 6 h after H-I reduced the number of TUNEL-positive cells by 70% and 64% respectively in CA1 and cortex compared to untreated H-I (*p* < 0.001); the application of NAAG decreased the number of apoptotic cells by 64% and 57% in the CA1 and cortex respectively, showing slightly weaker neuroprotective effect (*p* < 0.05 for both).

### Group II mGluR agonists reduce ROS levels after hypoxia-ischemia

**Fig 4:**
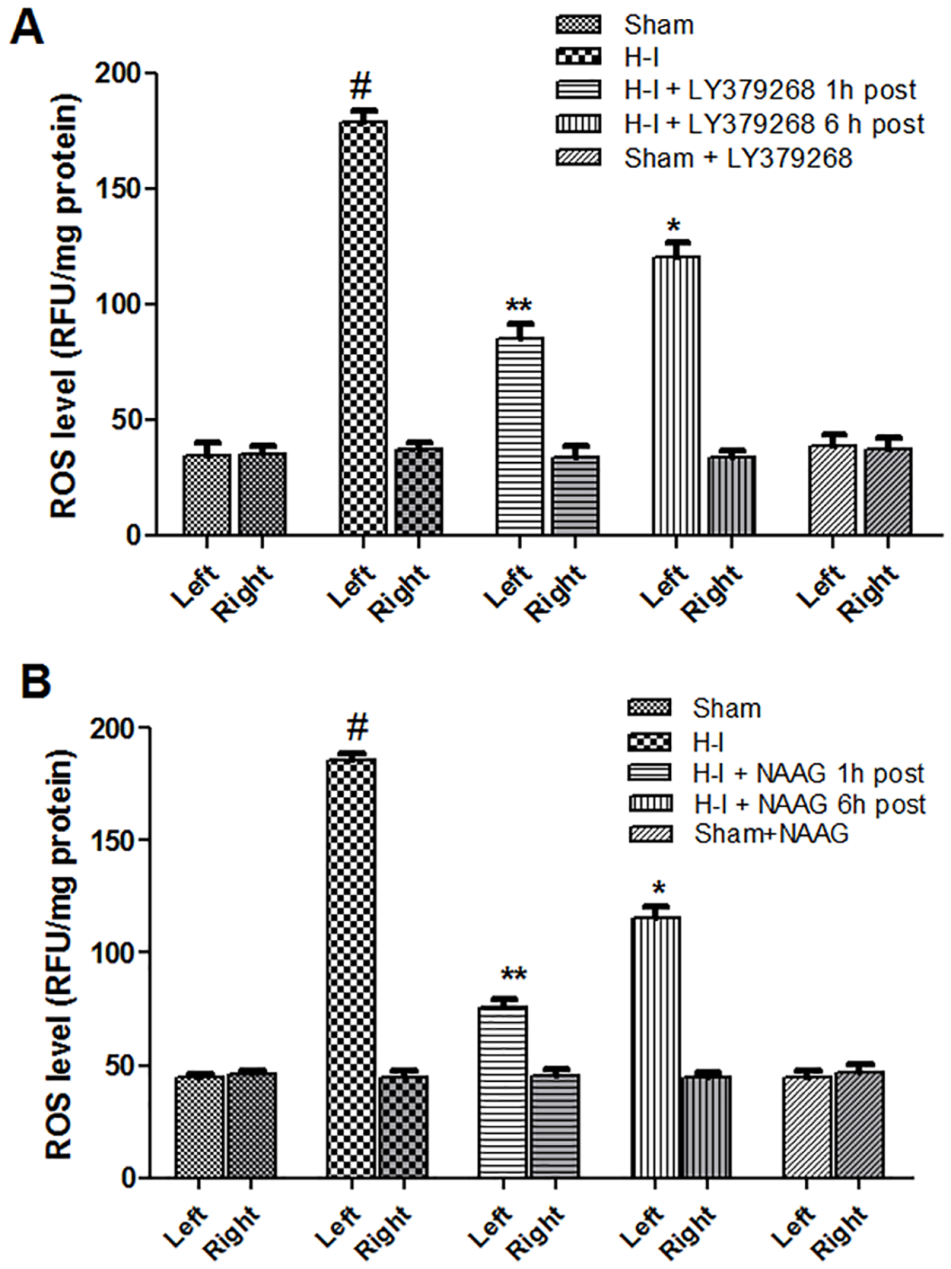
The effect of LY379268 (A) or NAAG (B) application 1 h or 6 h after H-I on ROS level. Results are presented as mean ± SEM, n = 3–4; #—different from the sham operated group, *p* < 0.001; different from H—I group: *- *p* < 0.005, **—*p* < 0.002.

The ROS level was measured in both hemispheres 3 h after the agonist injection. H—I increased the level of ROS in the ischemic hemisphere more than 4 times (F_2,6_ = 1650: *p* < 0.001) (Fig 4). LY379268 applied 1h or 6 h after H-I insult significantly decreased the ROS level by 52% and 33% respectively, compared to that observed after H-I (p < 0.002 and p < 0.005)(Fig 4a). The application of NAAG 1 h or 6 h after H-I also significantly suppressed increases in ROS production that resulted from the H-I, by 60% and 38% respectively (p < 0.001 in both cases)(Fig 4b). We found no statistically significant differences in the ROS level reduction between the agonists; however the decrease in ROS production observed when agonists were applied 1 h after H-I was significantly greater than the effect of application 6 h after H-I (p < 0.05).

Injections of LY379268 and NAAG did not affect the level of ROS in the sham operated animals, also neither H-I nor the injection of agonists changed ROS level in the right hemispheres.

### The effect of group II mGluR agonists on antioxidant enzymes activity and GSH concentration

**Fig 5:**
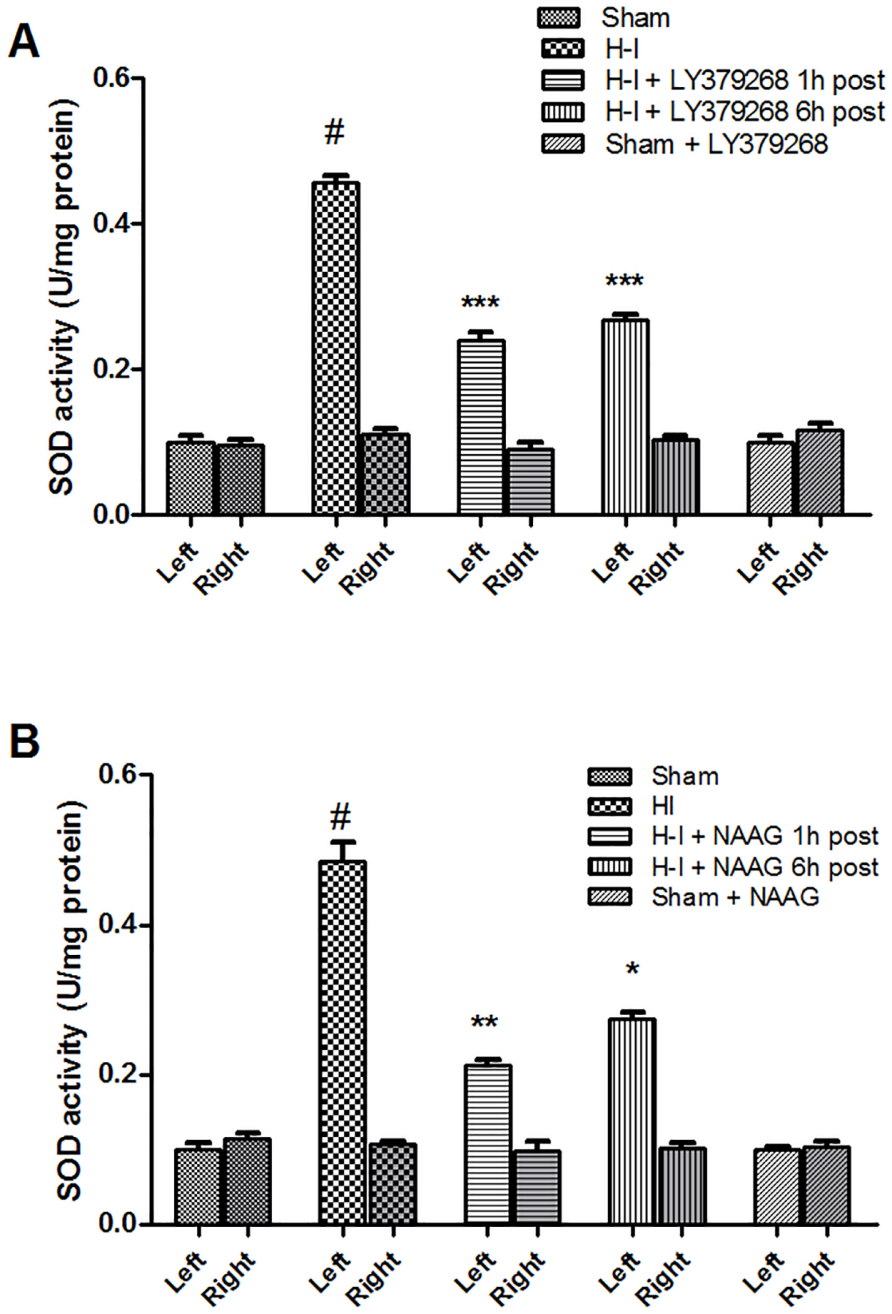
The effect of LY379268 (A) or NAAG (B) application 1 h or 6 h after H-I on changes in SOD activity. Results are presented as mean ± SEM, n = 4; #—different from sham operated group, *p* < 0.001; different from H—I group: *—*p* < 0.01, **—*p* < 0.005, ***—*p* < 0.001.

SOD activity was determined in both hemispheres 3 h after agonist injection. The SOD activity in the brain increased significantly after H-I to more than 400% of the control (F_3,12_ = 182,9; p < 0,001); however, this increase was observed only in the ischemic, ipsilateral hemisphere (Fig 5). The application of LY379268 1 h or 6 h after H-I resulted in a significant decrease in SOD activity, by 48% and 42% respectively, compared to the untreated H-I group (p < 0.001)(Fig 5a). Similarly, the injection of NAAG 1 h or 6 h after H-I reduced the SOD activity by 56% and 43% respectively (p < 0.005 and p < 0.01)(Fig 5b).

There was no statistically significant difference in the SOD activity reduction between the mGluR2 and mGluR3 agonists application; however, the decrease in SOD activity observed when agonists were applied 1 h after H-I was significantly greater than when agonists were applied 6 h after H-I (p < 0.001).

We observed no changes in SOD activity in the contralateral hemisphere after H-I, or after the injection of the agonists into the sham operated animals.

**Fig 6:**
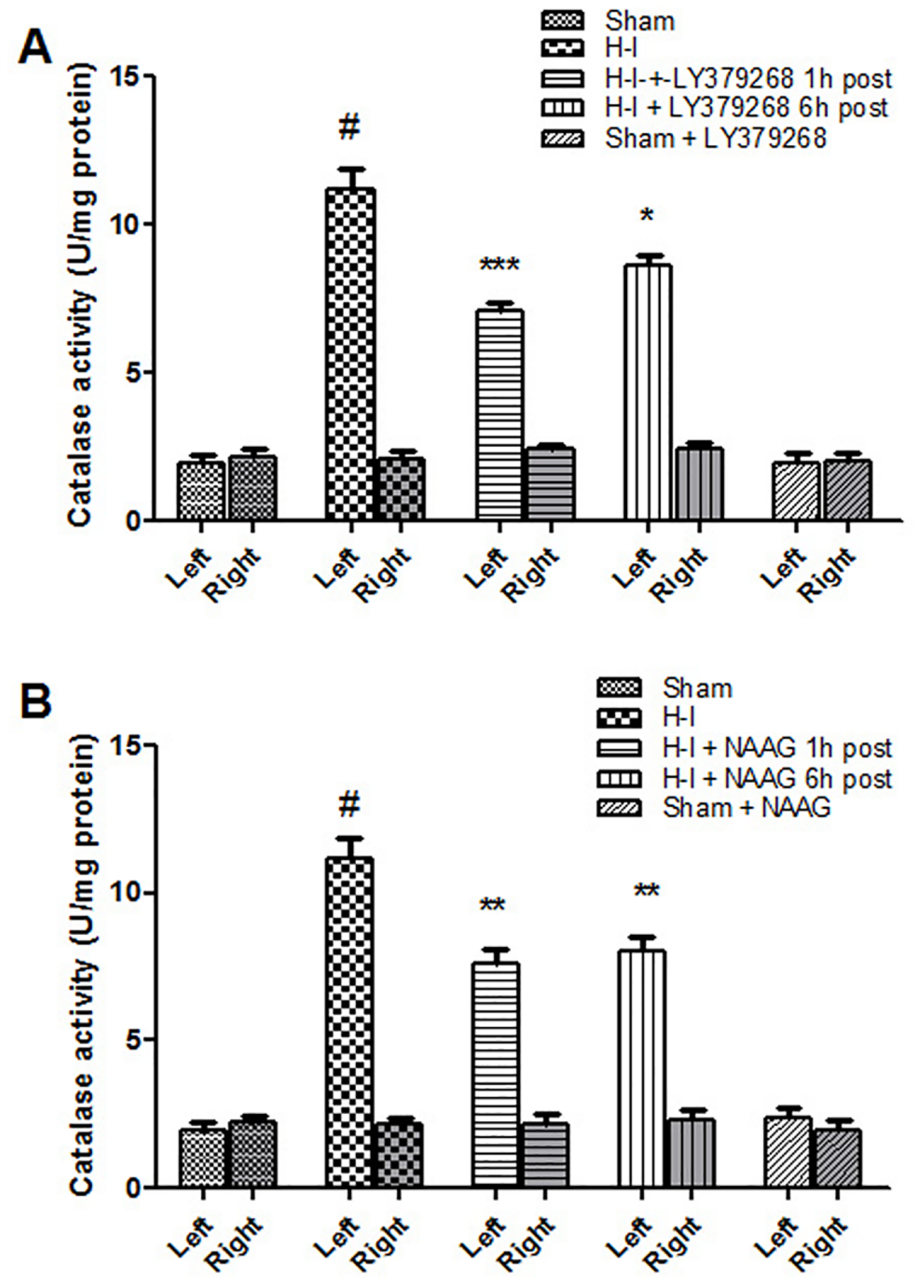
The effect of LY379268 (A) or NAAG (B) application 1 h or 6 h after H-I on catalase activity. Results are presented as mean ± SEM, n = 3–4; #—different from sham operated group, *p* < 0.001, different from H—I group: *—p<0.05, **—p < 0.005, ***—p < 0.001.

The activity of catalase also increased significantly in the ipsilateral hemisphere after H—I, reaching values five times higher than those of the control (F_1,6_ = 151; p<0,001) (Fig 6). LY379268 injected 1 h or 6 h after H-I significantly decreased catalase activity by 37% and 23% respectively compared to the untreated H-I (p < 0.005 and p < 0.05 respectively) (Fig 6a). The application of NAAG also significantly decreased the activity of catalase, by 32% (1 h) and 29% (6 h) (p < 0.02 in both cases) (Fig 6b). There was no statistically significant difference between the effects of mGluR2 and mGluR3 antagonists on catalase activity.

H—I did not change the catalase activity in the right hemisphere; also agonist application to the sham operated animals did not change the activity of catalase in young rat brains.

**Fig 7:**
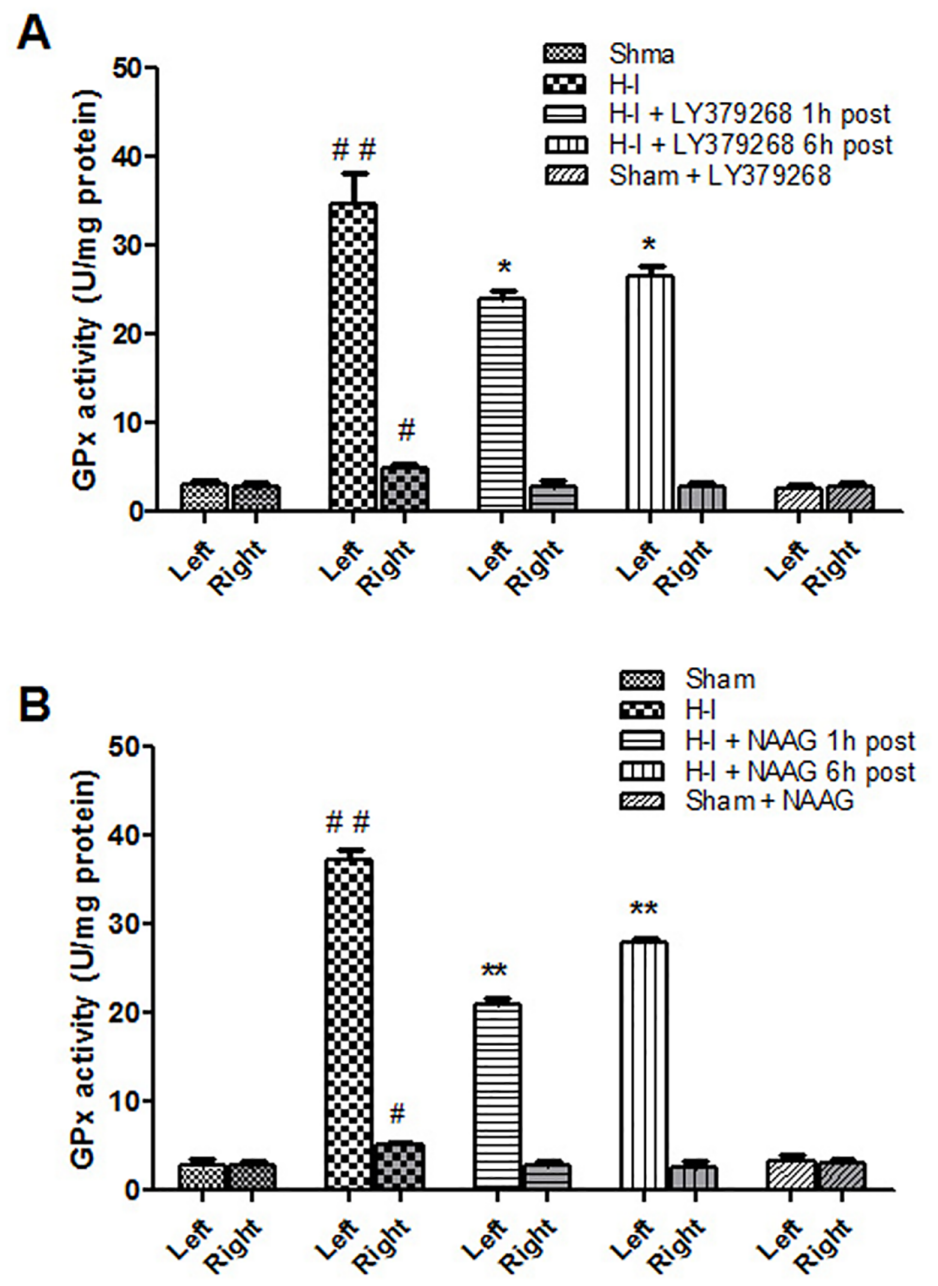
Changes in glutathione peroxidase activity observed after H-I and application of LY379268 (A) or NAAG (B) 1 h or 6 h after the insult. Results are presented as mean ± SEM, n = 3–4; #—different from sham operated group, *p* < 0.001; different from H—I group: *—*p* < 0.05, **—*p* < 0.001.

After H—I insult the activity of GPx, the second enzyme involved in anti H_2_O_2_ defense, in the left hemisphere increased more than 10 times in the left hemisphere (F_3,12_ = 109; *p* < 0.001). A slight but statistically significant increase in GPx activity was also detected in the right hemisphere (F_3,12_ = 7,89; *p* < 0.005) (Fig 7). The application of the mGluR2 agonist LY379268 1 h or 6 h after H-I insult significantly decreased the GPx activity in the left hemisphere by 31% and 24% respectively (p < 0.05 in both cases)(Fig 7a). The mGluR3 agonist, NAAG, injected 1h or 6 h after H-I also significantly decreased the GPx activity, by 44% and 25% in comparison to untreated H-I (p < 0.001 in both cases) (Fig 7b). In addition, the decrease in GPx activity observed after NAAG application 1 h after H-I was significantly greater than that observed from LY379268 after 1 h, or when NAAG was administered 6 h after H-I (*p* < 0.05 and *p* < 0.001 respectively). Each agonist reduced GPx activity in the right hemisphere to the control levels independently of the time of injection.

**Fig 8:**
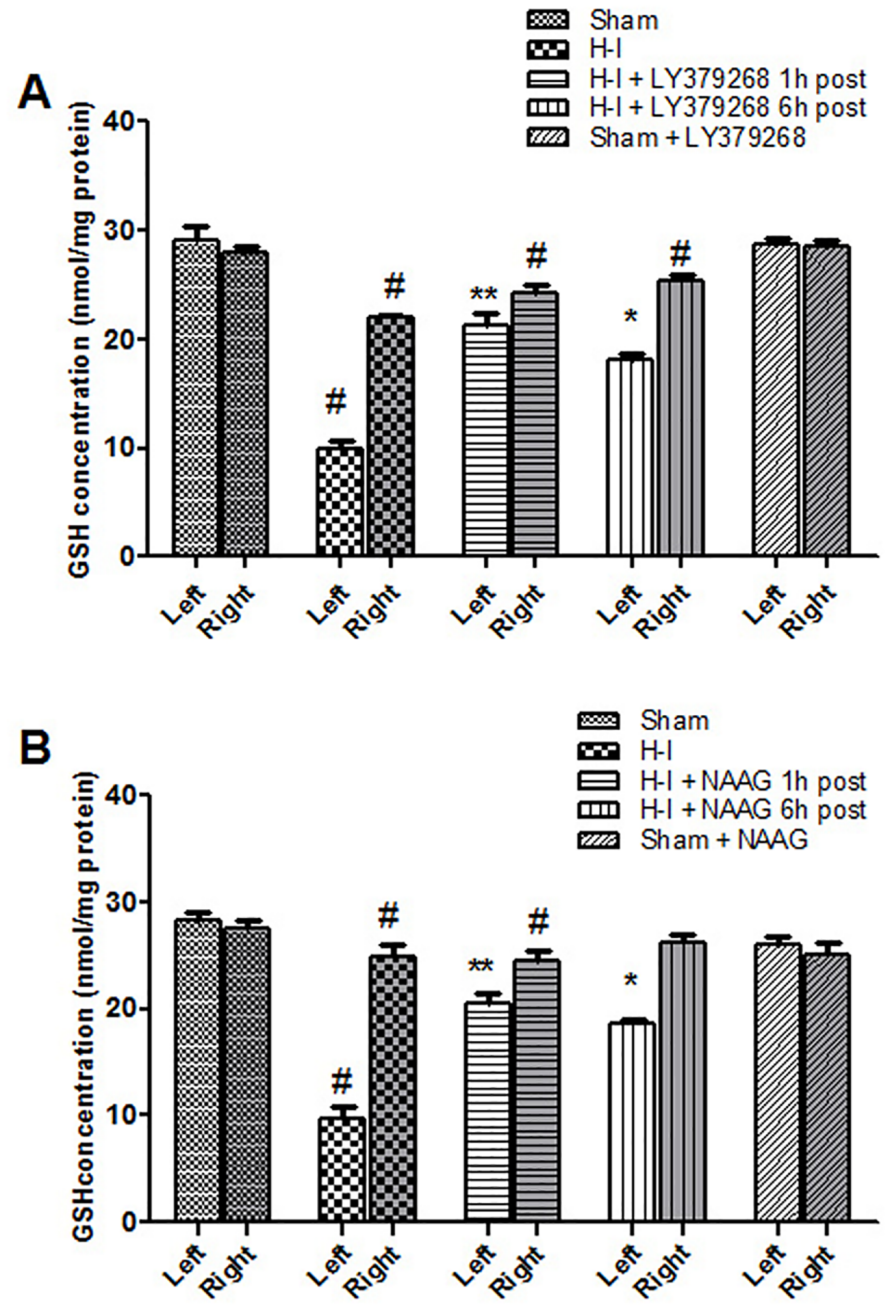
Partial restoration of GSH concentration by LY379268 (A) or NAAG (B) applied at different times after H—I. Results are presented as mean ± #—SEM, n = 4; #—different from sham operated group, *p* < 0.001; different from H—I group: *- *p* < 0.05, **—*p* < 0.005.

Glutathione content was measured in both hemispheres at time points corresponding to those used for the measurements of antioxidant enzymes. The GSH concentrations in brains isolated from control rats ranged between 28.6 ± 1.5 nmol/mg protein in the left and 27.7 ± 0.9 nmol/mg protein in the right hemisphere respectively. H-I resulted in a significant decrease in GSH concentration in the ipsilateral hemisphere to 34% of the control levels (F_3,12_ = 122.3; *p* < 0.001), with a less pronounced but statistically significant decrease to 84% of the control in the contralateral hemisphere (F_3,12_ = 14.9; p < 0.001)(Fig 8). LY379268 and NAAG applied 1h after H-I suppressed respective decreases in GSH concentrations to 73% and 72.9% of the control in the ipsilateral hemisphere, respectively (in both cases *p* < 0.001 compared to the untreated H-I); however, they had no effect on the GSH concentration in the right hemisphere (Fig 8a and 8b).

The application of agonists 6 h after H-I also inhibited the decrease in the GSH concentration, this time to 62.3% of control for LY379268 and 66% for NAAG (*p* < 0.005 in both cases); however, only NAAG restored the GSH concentration in the right hemisphere to that of the control. Interestingly, the effect of agonists applied 6 h after H-I on GSH concentration in the right brain hemisphere was more pronounced than when agonists were applied 1 h after insult.

## Discussion

Despite the long history of research conducted on birth asphyxia therapeutic hypothermia is the only treatment routinely used for clinical intervention. This approach does not ensure complete protection [29] and thus new therapeutics that can be safely used in clinical trials must be developed. Metabotropic glutamate receptors, such as group II mGluRs, are increasingly being considered as potential targets for neuroprotective drugs. The results of our experiments show for the first time that the neuroprotective effects of two group II mGluRs agonists on brain asphyxia are strongly related to the inhibition of ROS production. The application of mGluR2 or mGluR3 agonists (LY379368 or NAAG) in a short time after hypoxia-ischemia reduces the brain damage in the ipsilateral hemisphere. A decrease in ipsilateral hemisphere weight loss was also observed when agonists were applied 24 h after H-I. This finding reveals an expanded therapeutic window in which these drugs can be used; however, the mechanisms of the neuroprotective activity of mGluR2 and of mGluR3 agonists applied at such a long time after H-I may differ from those engaged in a short time after H-I.

Cerebral H-I in the newborns triggers a cascade of molecular consequences that begin with energy deficit. This cascade involves glutamate release, followed by subsequent glutamate receptors activation coupled with increased calcium influx, nitric oxide synthase activation and release of nitric oxide. This can result in mitochondrial dysfunction, generation and accumulation of ROS and initiation of oxidative stress [30,31]. Applications of LY3729268 or NAAG suppresses increases in ROS level evoked by an insult. The effects of using mGluR2 and mGluR3 agonists on ROS level and antioxidant enzymes activity are similar and do not differ statistically. However, despite these observations, the protective effects of LY379368 on neuronal damage are more pronounced than those of NAAG, especially when administered at longer time after H-I. The presented results also show that neuroprotective effects of both agonists are better expressed when agonists are applied 1 h after H-I.

A lack of selective agonists for group II mGluRs hampers the determination of the exact contribution of this group members to neurodegeneration. LY379268 is usually used as an highly selective agonist of mGluR2/3, with mGluR2 selectivity two times higher than mGluR3.

Additionally, as it was shown that in knock out mice where the lack of mGluR2 or mGluR3 is compensated by increased level of the remaining receptor, there was the lack of positive action of LY379268 in mGluR2 knock out mice in two mouse models predictive of antipsychotic activity. This finding suggests that it is the mGluR2 that mediates the actions of LY379268 and not mGluR3 [32,33].

Similarly, after several years of heated discussion, NAAG was finally accepted as a highly selective agonist of mGluR3 with only weak effects on NMDA receptors [34–37]. Therefore, in experiments presented in this paper we used LY379268 as an agonist of mGluR2 and NAAG as a specific mGluR3 agonist.

The neuroprotective effect of group II mGluRs agonists in ischemia has been observed through several experimental models. The application of NAAG to rats in a short time prior to, or after global and focal cerebral ischemia, resulted in significant neuroprotection [14,38]. Similar effects have been observed in a gerbil model of global ischemia for LY379268 and with another agonist which has a high affinity to mGluR2 –LY354740 [16,39]. The neuroprotective action of LY379268 and NAAG has also been reported in a neonatal rat hypoxia-ischemia model, but the mechanisms of their action have not been investigated in detail [13,15]. Folbergova et al. [21] have recently shown that the application of 2R,4R-APDC, an mGluR2/3 agonist, significantly reducs neurodegeneration and ROS production after DL-homocysteic acid seizures in immature rats.

H-I usually results in an increase in the activity of antioxidant enzymes SOD, catalase and GPx, and a decrease in GSH level. These changes are a result of the defensive mechanisms induced in the brain, which allows neurons to fight toxic concentrations of ROS. The results presented here show that the application mGluR2 and mGluR3 agonists in a short time after H-I reduces not only ROS levels but also the activity of antioxidant enzymes. This finding may suggest that the effect of mGluR2 and mGluR3 activation was directed towards the inhibition of ROS production rather than its removal. It is well established that the overexcitation of NMDA receptors is responsible for ROS production and that this is related to disturbances in mitochondrial functions, mainly of respiratory chain complexes I and III [40]. mGluR2/3 receptors, thanks to their ability to inhibit the presynaptic glutamate release in the brain, can attenuate excitatory synaptic transmission. Thus, it can be assumed that agonists of these receptors may suppress NMDA receptors stimulation and therefore ROS production. NAAG serves also as an endogenous modulator of NMDA receptors and attenuates their activation, which may represent an additional mechanism involved in the prevention of ROS production [41].

However, several other mechanisms of preventing ROS production, mainly by using mGluR3 agonist, have been proposed. Berent-Spillson and Russell [19] suggested that due to the increased synthesis of GSH in Schwann cells, the activation of glial mGluR3 protects neurons from oxidative injury resulting from high glucose concentration when in co-culture with Schwann cells.

GSH is an important cellular free radicals scavenger in both neurons and glia. GSH level has been shown to be higher in glial cells than in neurons, and it has been suggested that glia plays a pivotal role in maintaining antioxidant defense in neurons [24,42]. Our results show that the activation of both mGluR2 and mGluR3 restores decreased after H-I GSH concentration. However, based on experiments performed in our laboratory, it is difficult to determine whether the observed effect is related to increased GSH synthesis, or whether it is actually attributable to the prevention of its decrease.

Glutamate uptake by astrocytes is the main factor in the regulation of glutamatergic transmission and the maintenance of extracellular glutamate concentration below toxic levels. H-I significantly reduces glutamate uptake and this reduction is attenuated by the activation of astroglial mGlu3 and mGlu5 receptors [12,43,44]. Our results do not reveal differences in the neuroprotective effects between LY379268 and NAAG, which suggests that the main mechanism involved may be the inhibition of glutamate release. Both agonists were shown to decrease the extracellular glutamate concentration [14,45]. NAAG decreased extracellular glutamate concentration in an experimental model of focal cerebral ischemia in rat [14]. However, as mGluR3 are mainly expressed on astrocytes, we speculate that the neuroprotective effect of NAAG observed in our experiments may not be solely due to glutamate release inhibition but also be related to an increase in glutamate uptake by astrocytes.

The observation that the activation of group II mGluRs on glial cells results in the release of neurotrophic factor—transforming growth factor (TGF-α), which protects surrounding neurons from excitotoxicity [16,17,46], suggests that another mechanism is involved in the neuroprotective action of mGluR2/3 agonists. However, this pathway is not directly connected with ROS production and was not examined in the present study.

It was recently shown in a model of transient ischemia in rats, that the application of the mGluR2 positive allosteric modulator, LY487379, not only did not prevent damage to the CA1 region of the hippocampus, but also extends neuronal death to the CA3 region [47]. However, in these experiments, an agonist was applied 12 h after reperfusion, decreasing glutamate release at time when the activation of synaptic NMDA receptors containing the GluN2A subunit is necessary for effective neuroprotection [48,49]. In the experiments presented in this paper the mGluR2 agonist LY379268 was applied shortly after H-I (1 h and 6 h after H-I) and significant neuroprotection was observed in the CA1 region of the hippocampus and also in the cortex.

It has recently been shown that vulnerability to oxidative stress in neonatal rats subjected to H-I is dependent on sex. Sexual dimorphism has been observed in terms of mitochondrial functioning, antioxidant enzymes activity and behavioural outcomes [50,51]. However, the inhibition of respiratory chain activity has been observed in both sexes in similar way up to 18 h after an event [30] and the differences have been observed 20–24 h after H-I [52]. In our experiments, ROS level and antioxidant enzymes activity were measured 3 h after the injection of either of the agonists (up to 9 h after H-I). We did not find any differences that might be related to animals sex.

Our results show that both agonists of group II mGluR applied within a short time after H-I induce a decrease in ROS level after H-I whereas antioxidant enzyme activity remains similar to those of control values, clearly suggesting that the main neuroprotective mechanism observed involves the suppression of ROS production. The application of both, mGluR2 and mGluR3 agonists produced comparable results, indicating that both types of receptors may be serve as a potential target for future therapeutic action. Despite several potential mechanisms of ROS level reduction described in the literature, we note that the inhibition of excessive glutamate release and decrease in its extracellular concentration is the main mechanism involved in the observed patterns of neuroprotection.
